# Supplementary material for: Modelling the acclimation capacity of coral reefs to a warming ocean
Source: PLoS Comput Biol. 2022 May 9;18(5):e1010099. doi: 10.1371/journal.pcbi.1010099 (PMC9119535; doi:10.1371/journal.pcbi.1010099)
Supplement: S1 Appendix — (PDF) [file pcbi.1010099.s001.pdf]

S1 Appendix. Levels of bleaching

We collected literature data providing quantitative information about the reduction of symbiont abundance during laboratory-induced or natural bleaching events (Table A).

Table A: Literature sources from which we collected the data on the reduction of symbiont abundance during laboratory-induced or natural bleaching.

Location	Reference
Great Barrier Reef	[1–3]
South East Asia	[4–6]
the Caribbean	[7–11]

Fig A shows the reduction of symbiont abundance ΔS as a function of ΔT, the difference between the temperature at bleaching and the annual average temperature (for *in situ* observations) or the control temperature (for laboratory experiments). The data show a high variability in the reduction of symbiont abundance over a broad range of ΔT, in relation to both coral type (Fig Aa) and region (Fig Ab). This variation is likely the result of species-specific responses and the co-variation of temperature with other environmental factors. However, the overall range of percent reduction ΔS narrows down with increasing ΔT (Fig A). Therefore, we model bleaching as a random reduction of symbsiont abundance (within a uniform distribution constrained by ΔS_{obs}, the range observed in the data, Fig A) whenever environmental temperature T exceeds T^{opt}, the optimal temperature for coral growth (Fig 1b in main article), by an amount falling within the range of temprature variability (i.e., ΔT_{obs}, Fig A) observed during bleaching events.

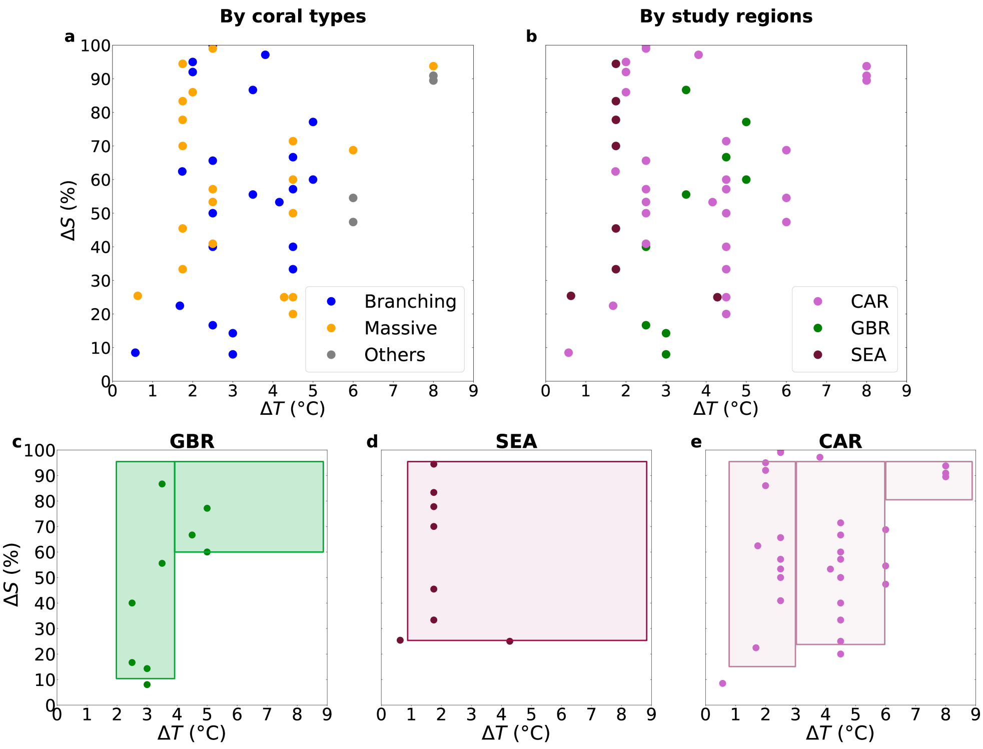

Fig A: Observations of percent reduction in symbiont abundance during bleaching events, by coral type (a) and by region (b). The coloured boxes (c,d,e) are guides to the eyes indicating the temperature bleaching range ΔT_{obs} (the width of the coloured boxes) and the corresponding symbiont abundance reduction range ΔS_{obs} (the height of the coloured boxes) used in our bleaching model (see Eq 11 and Table 2 in the main article).

References

1. Berkelmans R, van Oppen MJH. The role of zooxanthellae in the thermal tolerance of corals: a 'nugget of hope' for coral reefs in an era of climate change. Proceedings of the Royal Society B. 2006;273. doi:10.1098/rspb.2006.3567.

2. Hoegh-Guldberg O, Smith GJ. The effect of sudden changes in temperature, light and salinity on the population density and export of zooxanthellae from the reef corals Stylophora pistillata Esper and Seriatopora hystrix Dana. Journal of Experimental Marine Biology and Ecology. 1989;129(3):279–303. doi:10.1016/0022-0981(89)90109-3.

3. Jones RJ, Yellowlees D. Regulation and control of intracellular algae (= zooxanthellae) in hard corals. Philosophical Transactions of the Royal Society of London B: Biological Sciences. 1997;352(1352):457–468. doi:10.1098/rstb.1997.0033.

4. Brown BE, Le Tissier MDA, Bythell JC. Mechanisms of bleaching deduced from histological studies of reef corals sampled during a natural bleaching event. Marine Biology. 1995;122(4):655–663. doi:10.1007/BF00350687.

5. Brown BE, Dunne RP, Ambarsari I, Le Tissier MDA, Satapoomin U. Seasonal fluctuations in environmental factors and variations in symbiotic algae and chlorophyll pigments in four Indo-Pacific coral species. Marine Ecology Progress Series. 1999;191. doi:110.3354/meps191053.

6. Brown BE, Dunne RP. Solar radiation modulates bleaching and damage protection in a shallow water coral. Marine Ecology Progress Series. 2008;362. doi:10.3354/meps07439.

7. Glynn PW, D'Croz L. Experimental evidence for high temperature stress as the cause of El Niño-coincident coral mortality. Coral Reefs. 1990;8(4):181–191. doi:10.1007/BF00265009.

8. Fitt WK, Warner ME. Bleaching Patterns of Four Species of Caribbean Reef Corals. The Biological Bulletin. 1995;189(3):298–307. doi:10.2307/1542147.

9. Fitt WK, McFarland FK, Warner ME, Chilcoat GC. Seasonal patterns of tissue biomass and densities of symbiotic dinoflagellates in reef corals and relation to coral bleaching. Limnology and Oceanography. 2000;45(3):677–685. doi:10.4319/lo.2000.45.3.0677.

10. Hueerkamp C, Glynn PW, D'Croz L, Maté JL, Colley SB. Bleaching and Recovery of Five Eastern Pacific Corals in an El Niño-related Temperature Experiment. Bulletin of Marine Science. 2001;61(1):215–236.

11. Glynn PW, Maté JL, Baker AC, Calderón MO. Coral bleaching and mortality in panama and ecuador during the 19971998 El NiñoSouthern Oscillation Event: spatial/temporal patterns and comparisons with the 19821983 event. Bulletin of Marine Science. 2001;69(1):79–109.
