# Supplementary material for: Modelling the acclimation capacity of coral reefs to a warming ocean
Source: PLoS Comput Biol. 2022 May 9;18(5):e1010099. doi: 10.1371/journal.pcbi.1010099 (PMC9119535; doi:10.1371/journal.pcbi.1010099)
Supplement: S2 Appendix — (PDF) [file pcbi.1010099.s002.pdf]

S2 Appendix. Speed of acclimation

The speeds of acclimation N are constrained from observational data (Fig A). From simulations including bleaching events, we estimated $N = 5.54 \cdot 10^{-13}$, $N = 2.65 \cdot 10^{-13}$ and $N = 2.375 \cdot 10^{-13}$ for, respectively, the Great Barrier Reef, South East Asia and the Caribbean. As explained in the main article, these numbers are very small because, according to the units of the model, the speed of coral acclimation reflects the amount of energy that the equivalent of a cm^2 of coral cover (i.e., an extremely small portion of a coral colony) invests into the symbiotic relationship every month.

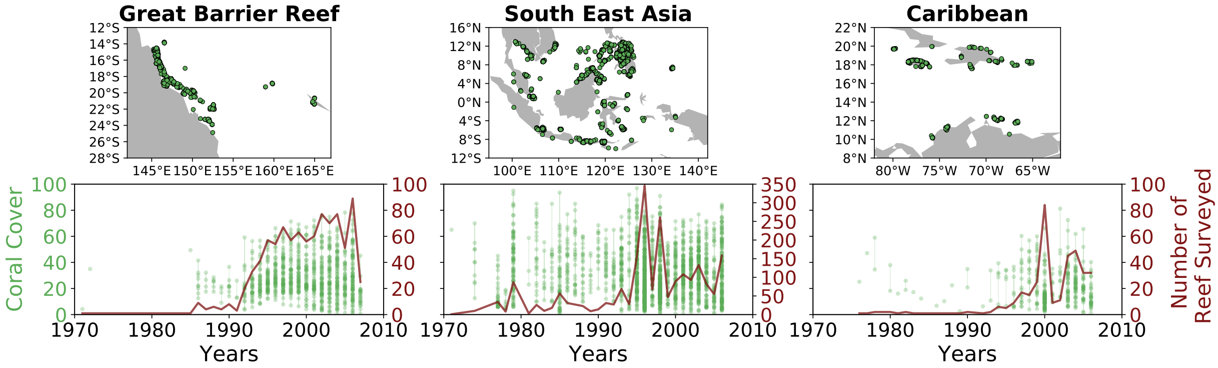

Fig A: Coral cover data. The green dots (top row) indicate all the reef sites that were surveyed between 1970 and 2006 in South East Asia and the Caribbean and between 1970 and 2007 in the Ggreat Barrier Reef. The light green dots (bottom row) are the coral cover data measured at a particular reef site, with thin green lines connecting the coral cover data collected in the same year and dark red lines indicating the number of reefs surveyed over the years. The reefs sites and the number of reefs surveyed were not fixed every year because the data were compiled from various literature sources until year 2007 [1]. The observational data in Fig 2 in main article are averages of all coral cover measured on all reefs surveyed at a given year (considering the mean values in the case of multiple measurements associated to the same reef name). The maps are produced with the Python's Matplotlib Basemap Toolkit available from `https://matplotlib.org/basemap/api/basemap_api.html` (© 2011, Jeffrey Whitaker).

References

1. Bruno JF, Selig ER. Regional decline of coral cover in the Indo-Pacific: Timing, extent, and subregional comparisons. PLOS ONE. 2007; p. e711. doi:10.1371/journal.pone.0000711.
