# Supplementary material for: Modelling the acclimation capacity of coral reefs to a warming ocean
Source: PLoS Comput Biol. 2022 May 9;18(5):e1010099. doi: 10.1371/journal.pcbi.1010099 (PMC9119535; doi:10.1371/journal.pcbi.1010099)
Supplement: S3 Appendix — (PDF) [file pcbi.1010099.s003.pdf]

S3 Appendix. Forcing data

Fig A shows the combined environmental temperature datasets (the WOD13 historical data from 1955 to 2010 and the future RCP scenarios from 2010 to 2100) used to force the model.

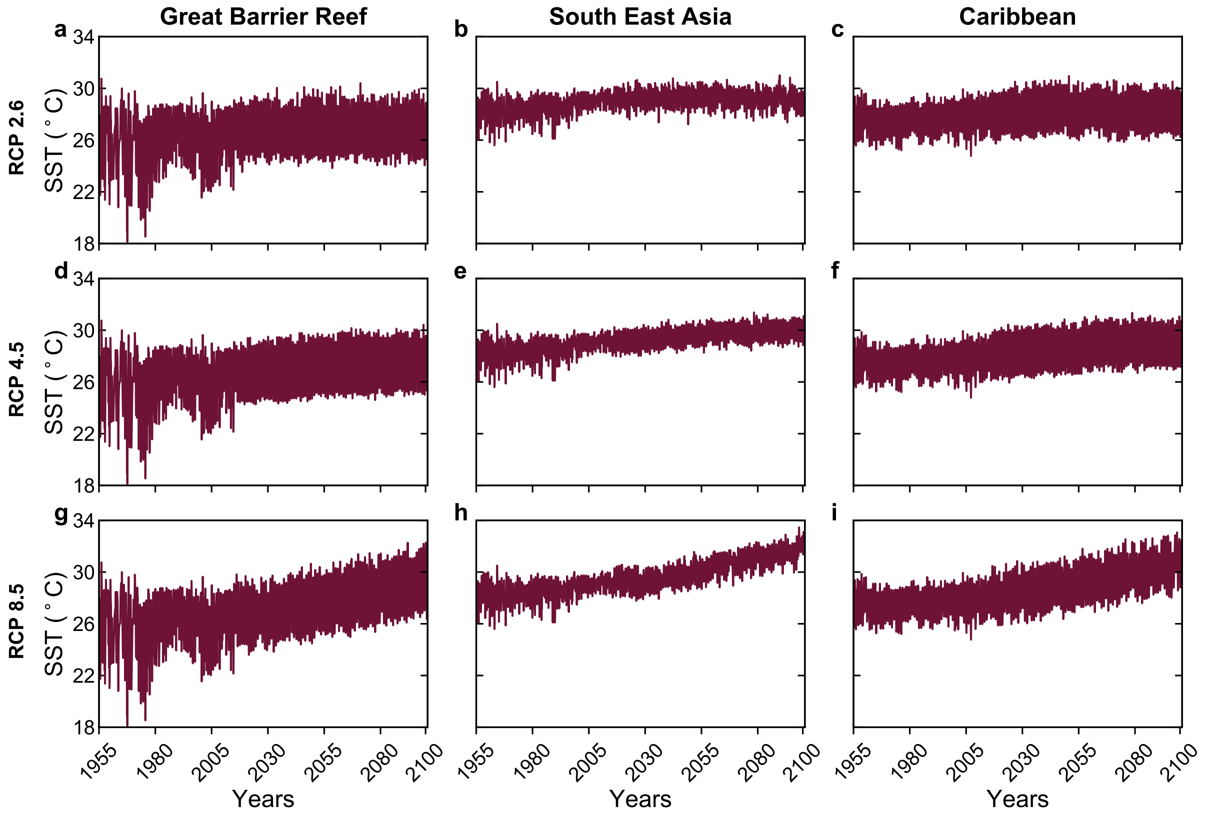

Fig A: Monthly Sea Surface Temperatures used as environmental temperature forcing (T in the model equations) for the model simulations that included bleaching. The datasets comprise historical temperature data (WOD13), from 1955 to 2010, and temperature projections (MPI Earth System Model), from 2010 to 2100.
