# Supplementary material for: Modelling the acclimation capacity of coral reefs to a warming ocean
Source: PLoS Comput Biol. 2022 May 9;18(5):e1010099. doi: 10.1371/journal.pcbi.1010099 (PMC9119535; doi:10.1371/journal.pcbi.1010099)
Supplement: S4 Appendix — (PDF) [file pcbi.1010099.s004.pdf]

S4 Appendix. Simulations without bleaching

We used the estimated speeds of coral acclimation N to determine the response of corals under the hypothetical absence of short-term (monthly) temperature fluctuations (i.e. without bleaching). This model experiment is conducted using annually averaged temperature forcing (Fig A). Due to a small offset appearing as a step change between the WOD13 and the RCP scenarios data, we introduced a correction to the data in order to match the two temperature time-series (Fig A).

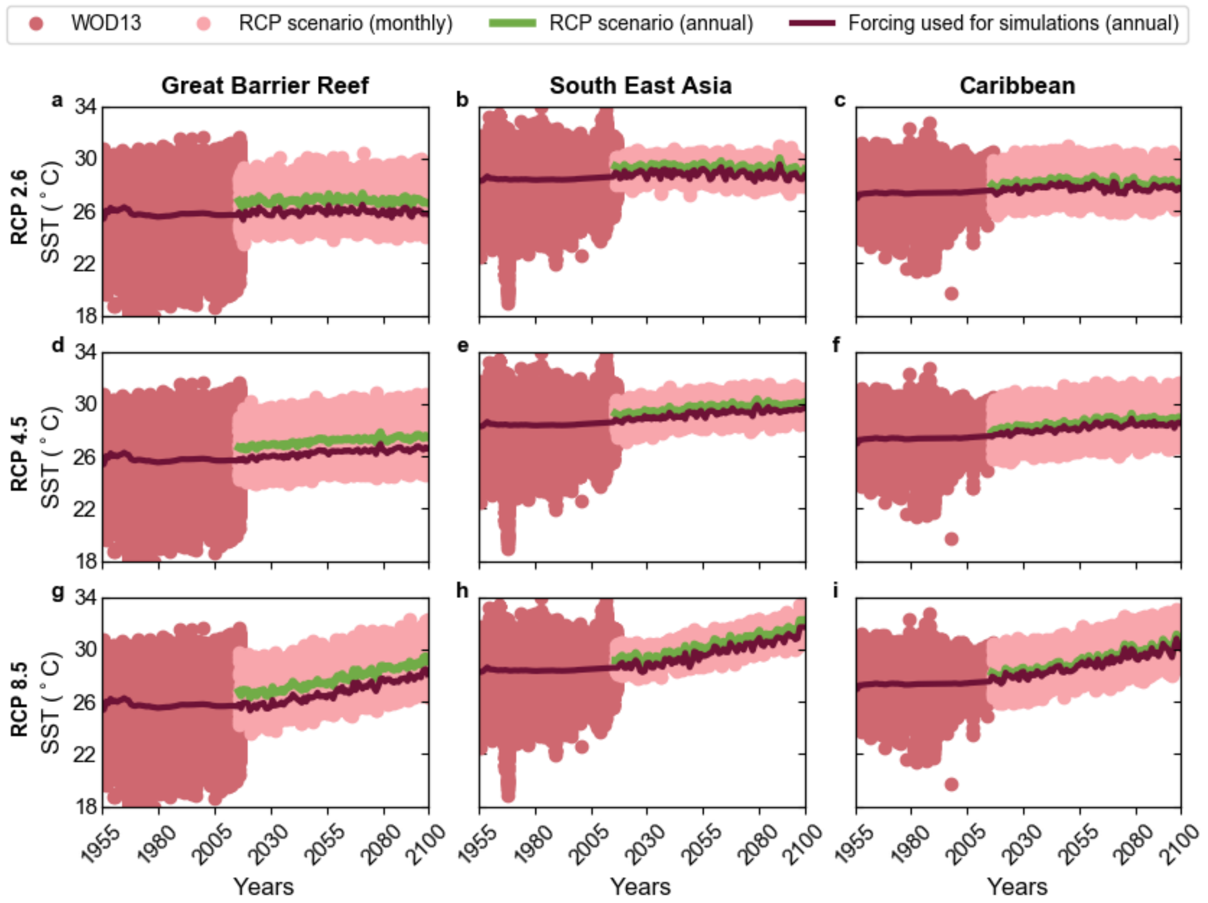

Fig A: Annual Sea Surface Temperatures used for the model simulations that excluded bleaching. The datasets comprise historical temperature data (WOD13, dark pink dots), monthly (light pink dots) and annual (green line) temperature projections produced by the MPI Earth System Model, and actual temperature data used as forcing (brown line, T in the model equations) with off-set between historical and projections data removed.

Fig Ba–c shows the temperature-limited coral growth rate and the temperatures at year 2015 and 2100 for the three emission scenarios. For the lowest emission scenario (RCP 2.6) and for all regions, there is no major difference between the temperature at year 2015 and the projected temperature for the year 2100. Big differences are noticeable between the temperature in 2015 and 2100 under moderate (RCP 4.5) and high (RCP 8.5) emission scenarios (Fig Ba–c). However, under RCP 4.5 and 8.5, temperatures for 2100 are within the coral thermal-limited growth curves for the Great Barrier Reef region. In South East Asia and in the Caribbean, temperatures for the year 2100 remain within the coral thermal-limited growth curves only under RCP 4.5.

When we exclude bleaching events, corals adapt towards higher energy investment

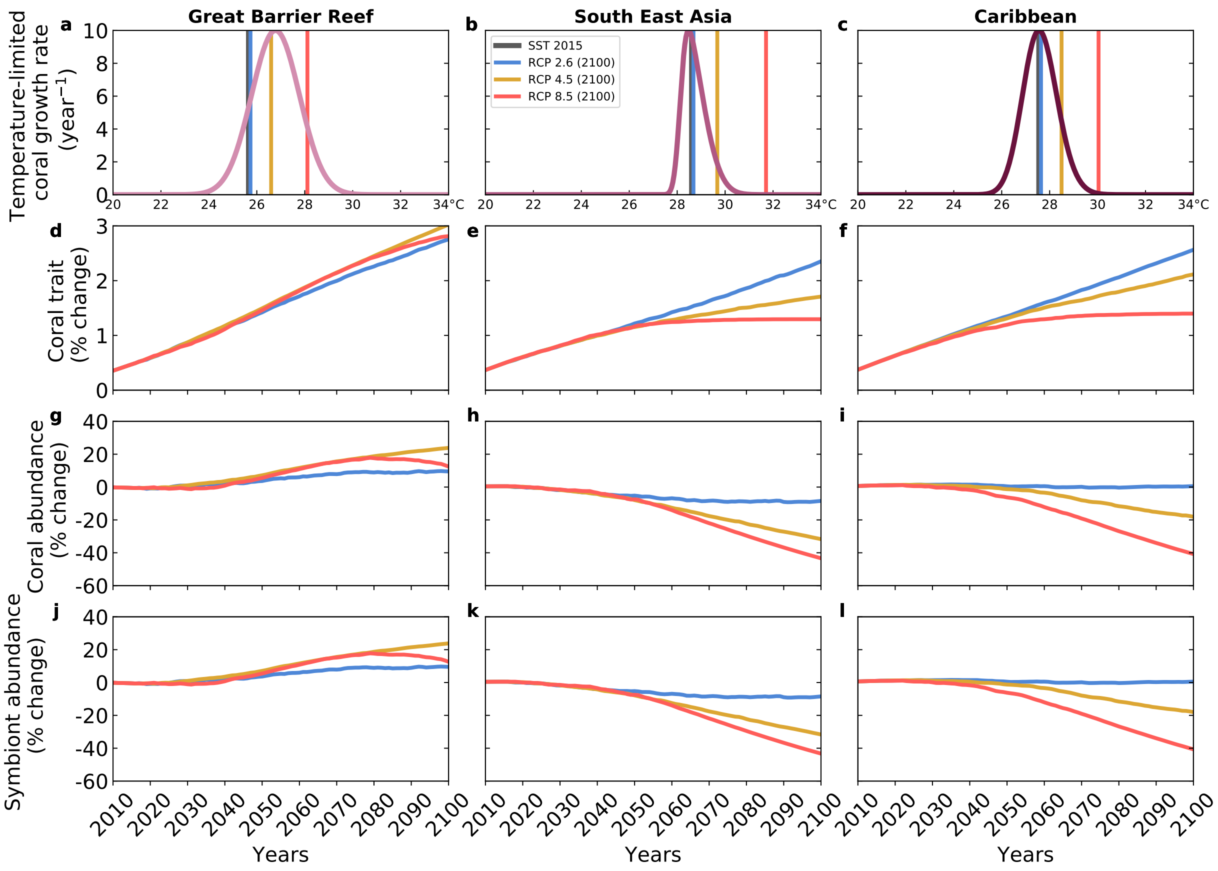

Fig B: Coral thermal-limited growth curves (**a-c**), with vertical lines indicating average temperatures at different years and for different RCP scenarios. Dynamics of trait (**d-f**), coral abundance (**g-i**), and symbiont abundance (**j-l**) relative to the period 1986-2005 and excluding bleaching.

traits regardless of region and emission scenario (Fig Bd–f). This illustrates the corals' ability, in our model, to adjust to higher trait value in order to compensate for the growth deficiency induced by increasing temperature. In all regions, RCP 2.6 does not produce appreciable changes in coral abundance (Fig Bg–i). In the Great Barrier Reef, coral abundance starts increasing around the year 2040 under both RCP 4.5 and RCP 8.5 because environmental temperatures increase towards the optimal temperature for coral growth (Fig Ba). In South East Asia and the Caribbean, coral abundances start decreasing from around year 2040 under both RCP 4.5 and RCP 8.5 because environmental temperatures moves beyond the optimal temperature for coral growth (Fig Bh–i). The dynamics of the symbiont abundance is similar to that of the coral abundance in all scenarios (Fig Bg–l) because symbionts grow logistically (Eq 2) with carrying capacity proportional to coral abundance (Eq 10) and because bleaching is excluded.

For the period 2081-2100 relative to 1986-2005, our model produces declines in coral abundances by 27 % and 37 % under, respectively, RCP 4.5 and RCP 8.5, in South East Asia; and declines in coral abundance by 15 % and 34 % under, respectively, RCP 4.5 and RCP 8.5, in the Caribbean.
