# Supplementary material for: Modelling the acclimation capacity of coral reefs to a warming ocean
Source: PLoS Comput Biol. 2022 May 9;18(5):e1010099. doi: 10.1371/journal.pcbi.1010099 (PMC9119535; doi:10.1371/journal.pcbi.1010099)
Supplement: S5 Appendix — (PDF) [file pcbi.1010099.s005.pdf]

S5 Appendix. Sensitivity analysis

We performed a sensitivity analysis using the mean coral abundance between the years 2000 and 2100 as a reference. We deem it sufficient to look at the sensitivity of coral abundance only because the persistence of coral reefs depends mainly on the survival of corals.

Sensitivity to the speed of acclimation. We performed simulations over a range of speeds of acclimation with and without bleaching. We scaled the resulting coral mean abundances in order to highlight the effects produced by the changes in parameter values. In all regions, coral abundance increases with increasing speed of acclimation under $\pm 25\,\%$ change in each parameter (Fig A). This indicates that our acclimation formulation is robust with respect to any parameter change because coral abundance always increases with increasing speed of acclimation, as expected, given that faster acclimation should always lead to higher abundance yield.

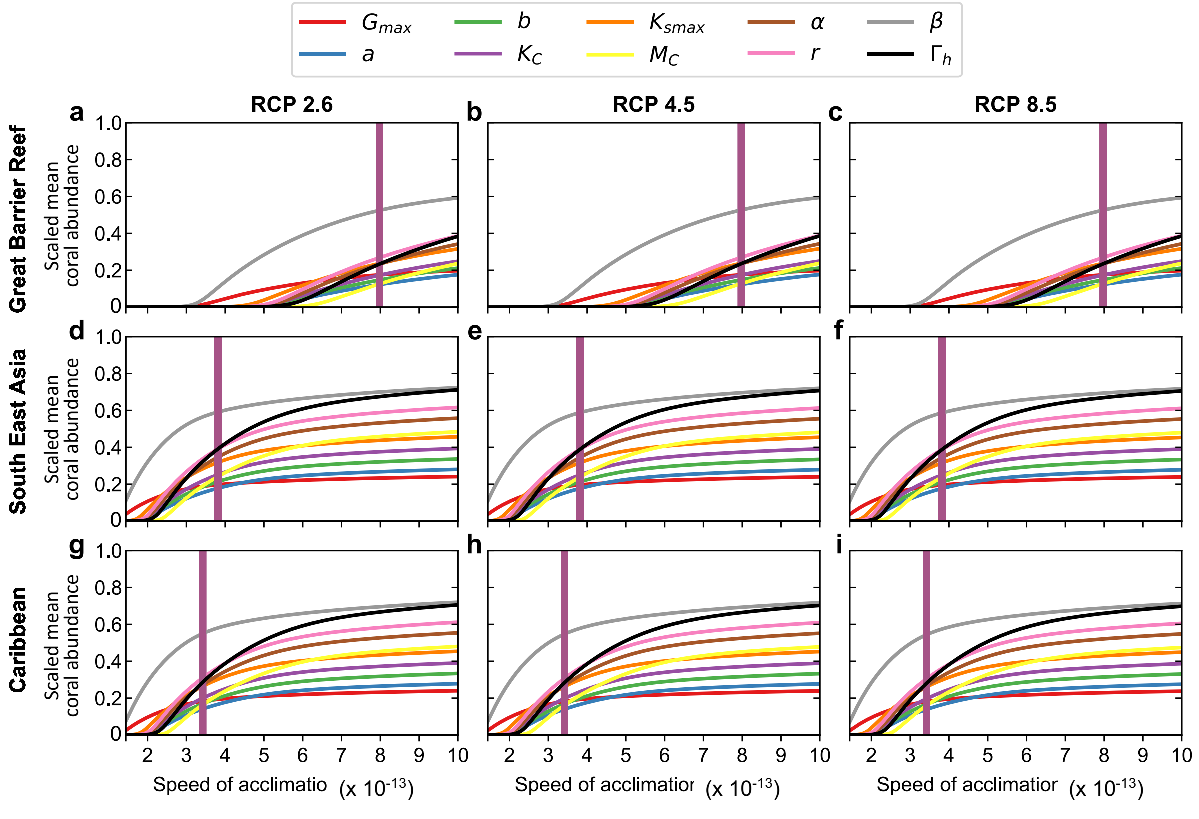

Fig A: Sensitivity to speed of acclimation N (simulations without bleaching) for $+25\,\%$ change in each parameter (indicated in the legend). Vertical lines mark the speed of acclimation we estimated from coral cover data (see S2 Appendix).

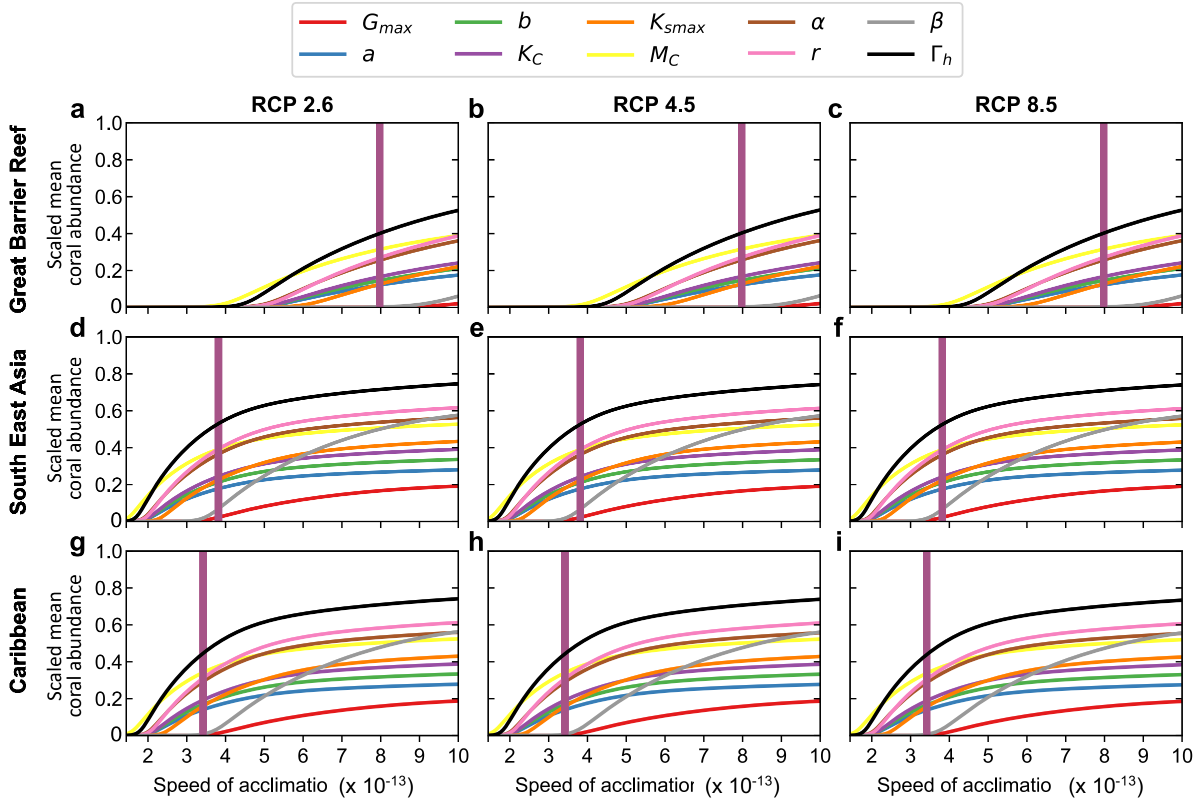

Fig B: Sensitivity to speed of acclimation N (simulations without bleaching) for $-25\,\%$ change in each parameter (indicated in the legend). Vertical lines mark the speed of acclimation we estimated from coral cover data (see S2 Appendix).

Sensitivity to all parameters. For these sensitivity tests, we used the following formula

$$100 \cdot \left(\frac{C_{\text{sens}} - C_{\text{standard}}}{C_{\text{standard}}} \right),$$

where C_{sens} is the mean coral abundance between 2000 and 2100 obtained with $\pm 25\,\%$ change in a given parameter and C_{standard} is the mean coral abundance between 2000 and 2100 obtained with the standard value of the given parameter (Table 1 in main article). Parameters were changed one at a time, while keeping all the others fixed to their standard values.

In all regions and regardless of the emission scenario, the model without bleaching shows that a $+25\,\%$ change in maximum coral growth rate (G_{max}), coral carrying capacity (K_C), and strength of symbiotic feedback (β), produced positive changes in coral abundance (Fig C). In contrast, a $-25\,\%$ change in the same parameters produced negative changes in coral abundance. These results are consistent with our model formulation because higher (lower) values of these parameters should increase (decrease) coral fitness by increasing (decreasing) both coral growth (Eq 4) and gains via the symbiotic feedback (Eq 5). Increase (decrease) in coral abundance is consistently produced when coral mortality (M_C) is decreased (increased) by $25\,\%$.

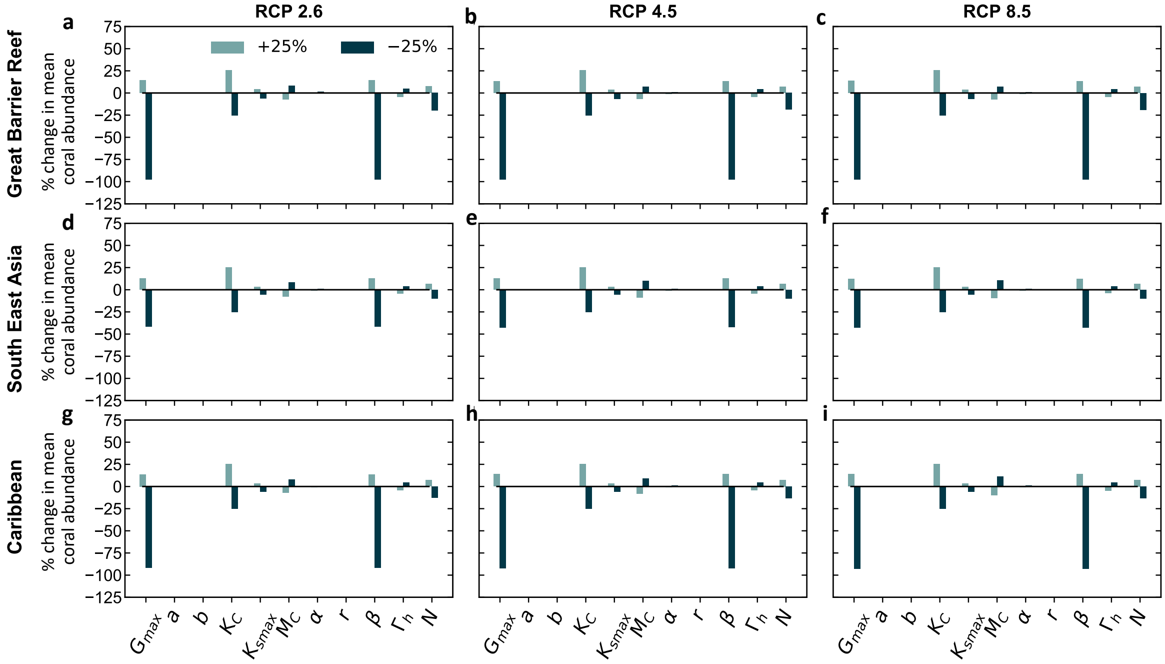

Fig C: Percent difference in mean coral abundance to $\pm\,25\,\%$ variations in each parameter for the model without bleaching.

In general, the model with bleaching shows higher sensitivities than the model without bleaching. More specifically, the model results with bleaching for the the Great Barrier Reef (Fig Da-c) and the Caribbean (Fig Dd-f) appear more sensitive than those for South East Asia (Fig Dg-i) for the $+25\,\%$ change. For example, a $+25\,\%$ increase in maximum coral growth rate (G_{max}) produces a percent change in coral abundance of only $30\,\%$ in South East Asia (Fig Dd), as compared to $80\,\%$ change in the Caribbean (Fig Dg). This is because the higher number of bleaching events occurring in South East Asia reduces the sensitivity of coral abundance to G_{max} due to a reduced symbiotic feedback following the recurrent expulsions of symbionts.

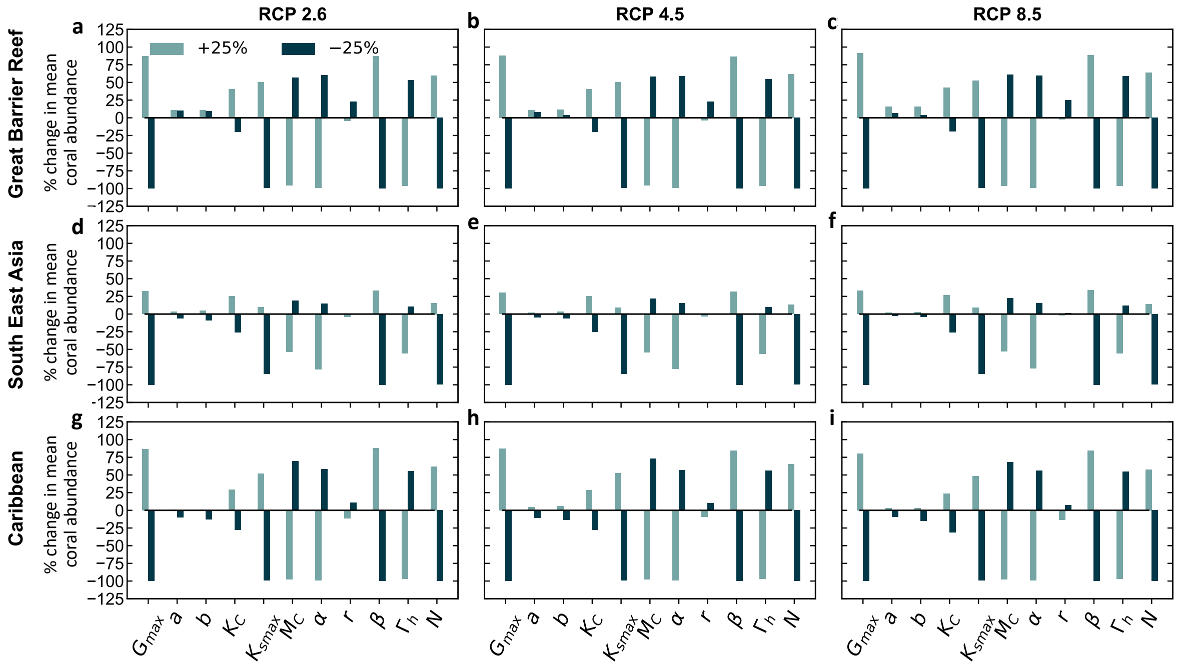

Fig D: Percent difference in mean coral abundance to ± 25 % variations in each parameter for the model with bleaching.
