# Supplementary material for: Modelling the acclimation capacity of coral reefs to a warming ocean
Source: PLoS Comput Biol. 2022 May 9;18(5):e1010099. doi: 10.1371/journal.pcbi.1010099 (PMC9119535; doi:10.1371/journal.pcbi.1010099)
Supplement: S6 Appendix — (PDF) [file pcbi.1010099.s006.pdf]

# S6 Appendix. Symbiotic relationship: benefits versus costs for corals

To illustrate the relative contribution of benefits obtained and costs sustained by the corals in the symbiotic relationship, we present here the two variable contributions to coral fitness: coral gross growth (reflecting the benefits) and costs of symbiosis (Fig A). This figure is produced with the model results presented in Fig 3 in the main article. Both these components contribute to coral fitness (Eq 4). The costs of symbiosis (reflecting the investments of corals into the symbiotic relationship) follow closely the trajectory of the benefits received by the corals from the symbiotic relationship. When such benefits are minimal also investments are minimal and vice versa.

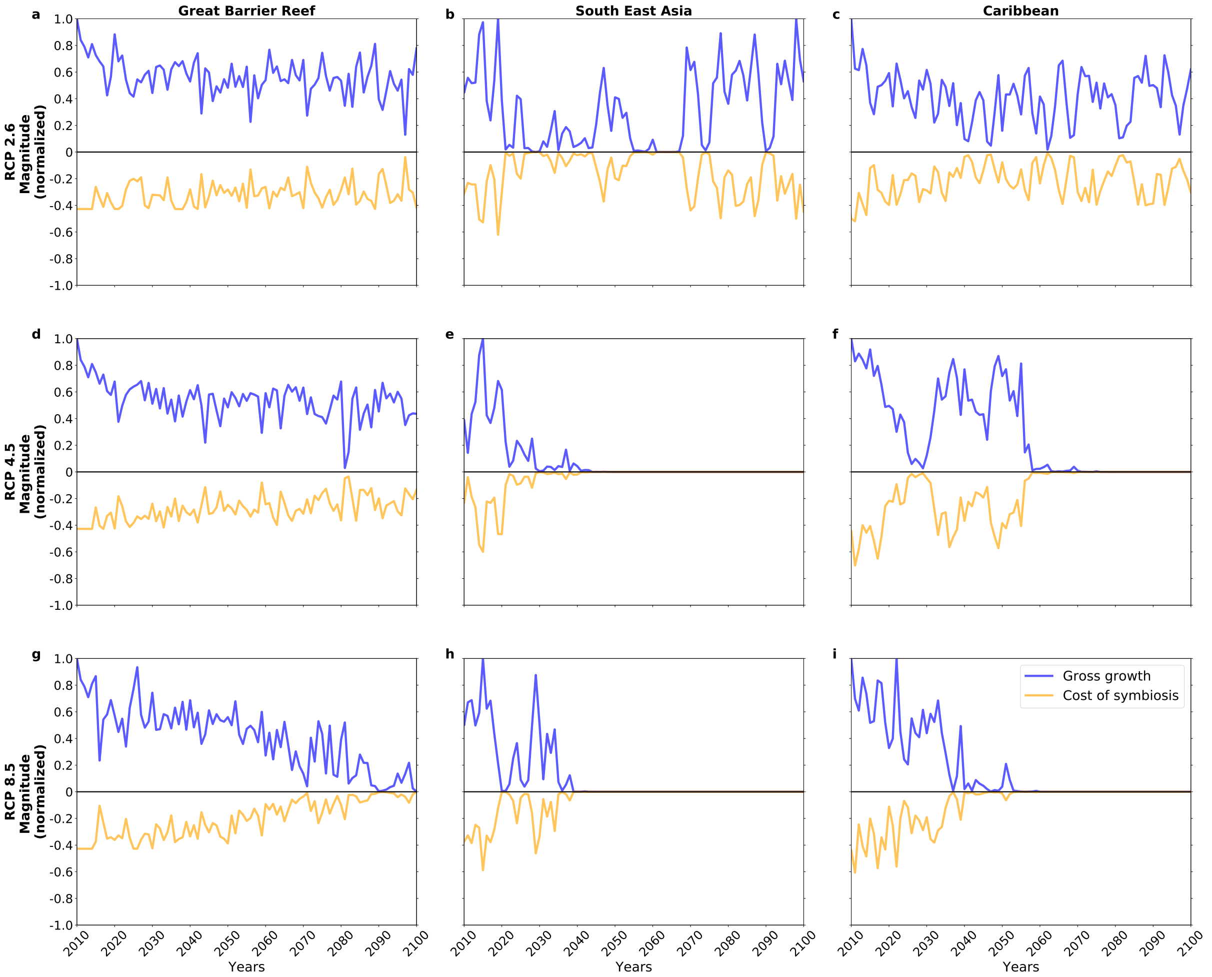


**Fig A:** Coral gross growth (blue lines), reflecting the benefits received by the corals from the symbiotic relationship (positive term in Eq 4) and costs (orange lines), reflecting the investments made by the corals for maintaining the symbiotic relationship (first negative term in Eq 4).
