# Supplementary material for: Modelling the acclimation capacity of coral reefs to a warming ocean
Source: PLoS Comput Biol. 2022 May 9;18(5):e1010099. doi: 10.1371/journal.pcbi.1010099 (PMC9119535; doi:10.1371/journal.pcbi.1010099)
Supplement: Attachment — Submitted filename: Responses20220303.pdf [file pcbi.1010099.s007.pdf]

Point-by-point replies to reviewers

We appreciate the reviewers' constructive feedback and the many suggestions received. They have helped us to strengthen our manuscript both scientifically and in terms of clarity of meaning. Below we respond, point-by-point, to the reviewers' comments (quoted in italics) and we specify the revisions implemented in the manuscript. Please note that, for a more effective and easier assessment of our revisions, the lines we cite in the responses below refer to the revised manuscript with tracked changes. Please, note also that, to comply with the journal policy, we uploaded the appendices of the manuscript as separate items of Supplementary Information. However, in the version of the manuscript with tracked changes, the appendices are still there for an easier and quicker assessment of the changes we included in them.

Reviewer 1

In this paper, the authors develop a computational model of coral-symbiont dynamics in the context of rising sea surface temperatures to estimate the extent to which phenotypic plasticity can keep pace with global warming projections in three distinct coral reef systems: the GBR, SE Asia, and the Caribbean. The authors first formulated a general dynamical population model and then visually fitted predictions of this model to multi-decadal time series of coral cover in each of the three focal reef systems. This fitting provided system-specific estimates for rates of adaptation in a coral trait that translates to energetic investment in symbiotic zooxanthellae, estimates then used in future projections of long-term coral cover in each system under each of three region-specific future climatic warming projections. The authors find that the ability of coral communities to acclimate to future warming is generally limited but varies by region, with the GBR showing the greatest and the Caribbean the least capacity to adapt to keep pace with climatic projections. This work contributes a novel, empirically rooted computational modeling framework that provides important initial insights on the capacity for reef forming corals to use phenotypic plasticity to counter warming - a poorly understood phenomenon that is fundamental to our understanding of the ecology and conservation of coral reefs globally in the face of unprecedented anthropogenic threats.

I really enjoyed reading this paper. Aside from very minor grammatical issues throughout, it was easy to follow and was well motivated, methods and results clearly presented, and conclusions well justified by the findings. I also think that this type of global scale, data-motivated projection modeling is exactly the kind of work that is needed to set the stage to establish realistic, empirically rooted estimates for what the future will hold for critically threatened ecosystems. I commend the authors on the novelty and practicality of the work. I believe that a revised version of this work could be a highly cited addition to this journal. To this end, below I offer some comments and suggestions (with line number, where applicable) that I hope held improve the final version of the ms.

Thank you for the positive words about our study. We regret that you found some minor grammatical issues. We carefully revised the manuscript to improve grammar and readability.

First, since coral cover is the only metric used in the time series that the model is being fitted to, I find myself wondering how much a signal of acclimation could be falsely attributed to a shift in coral species assemblage. For example, if temperatures increase over, say, 5 years in one of the study regions, and coral cover initially dips but then rebounds, this may fit the results of a model with acclimation and no species turnover when it is actually species turnover in this case that is driving the pattern. I appreciate that the authors qualify their findings in several instances by acknowledging both that this work is essentially a 'first pass' at estimating a very difficult, but crucial (from a conservation perspective) empirical metric (acclimation rate) and that other, not-modeled factors could be playing a significant role in the time series to which the model was fitted. But I think the authors could go a bit deeper here (e.g., discussing leading alternative drivers for the patterns observed and the likelihood that these would quantitatively or qualitatively affect the paper's conclusions), to better contextualize their findings and the extent to which it seems likely that the model's estimates for acclimation rate would be anywhere near reality. This additional content would also help paint a clearer picture for future work, by helping to prioritize the most timely extensions of this modeling framework.

Thank you for pointing this out. Our model does not resolve the species-specific mechanisms that may drive changes in the trait *U* (which is intended as a community-aggregate trait). Changes in this trait may reflect physiological processes and/or ecological processes. In turn, such processes may be brought about by shifts in species composition. The species composition changes and, with it, the "average" physiology of the whole community, for example in terms of the average sensitivity of the

community to temperature. Our aim is to track down such mean changes through the trait *U*. Obviously, this approach misses species-specific information but it does capture the average effects of species-specific changes at the community level. We clarified these aspects in the revised manuscript in different places. In the Abstract, we specified that the speed of coral acclimation is estimated at the community level. In the Introduction, we clarified that our new model captures a whole coral community by considering the average energy invested in the symbiotic relationship as a mean community trait fuelled by the coral's physiological machinery (lines 47 – 49). More crucially, in the Discussion we explained that physiological acclimation in our model encompasses phenotypic changes that could be brought about by plastic changes in the physiology of the different populations composing the coral community or by shifts in species compositions and that, despite our model does not resolve single species, the changes in the trait captured by the model reflect the effects that such shifts may have at the community level (lines 425 – 429). Additionally, we specified, wherever relevant in the manuscript, that the trait is a community-average trait that captures the coral community as a single acclimating entity.

Second, I think it would be helpful to have a bit more motivation for the mechanism of acclimation - how realistic is it for trait-dependent increases in coral energetic investment in symbionts to prevent bleaching? And what occurs physiologically to allow this to happen? A deeper foray into why this approach is realistic would help, I think, give credence to the overall paper results, which stem from these fundamental assumptions.

Thank you for this comment. In the original manuscript, we actually described possible realistic mechanisms of acclimation (lines 41 – 44 and lines 198 – 201, in original manuscript). For additional clarity, at the beginning of the Discussion, where we mention again that many different physiological and ecological mechanisms could be involved in coral acclimation, we now cite the relevant publications (line 421) in which specialised readers can find additional information on such mechanisms. The additions in the Discussion related to the shifts in species composition implemented in response of your point above (lines 425 – 429) are further clarifications about the mechanisms of acclimation. We hope that our original text and these revisions are sufficient for the reader to form an idea about the plausibility of our approach.

More specific / minor commoners:

Fig. 2 shows data juxtaposed to simulations for different acclimation rates, but it would be helpful to contextualize these plots a bit more by also showing SST over this same time period, perhaps absolute temps with a reference to each study region's optimum value from Fig. 1b.

Thank you for this comment, we followed your suggestions by adding SSTs relative to corresponding regional optima as thin grey lines (see new Fig. 2). Accordingly, we also revised the caption of Fig. 2 by explaining what these grey lines are.

171: "We implement bleaching as a reduction in symbiont abundance when environmental temperature exceeds Topt, i.e. the optimal temperature for coral growth." Table 2 then mentions that once the threshold temperature is exceeded, bleaching is determined by a draw from a uniform distribution. But isn't bleaching severity correlated with temperature? Thus, I'm wondering: why not make bleaching a monotonic function of temperature, after the threshold is crossed, or, to maintain the stochastic element, make it a draw from a skewed distribution that reflects the bias of greater bleaching with greater temperatures? This seems reasonable, and so could be worth using, even as an alternative formulation to test the robustness of findings.

The bleaching parameters in Table 2 were actually estimated empirically from observational data of temperature-induced bleaching. These data are presented in S1 Appendix. Panels 'c', 'd', and 'e' of Figure S1 show the regional ranges of symbiont reduction following bleaching events from which we estimated the bleaching thresholds used in our model. To clarify this aspect, we added the following text in the caption of Table 2: "These ranges were obtained from observational data of temperature-induced bleaching (see Fig S1, for additional details)". Other changes related to this aspect include:

- Table S1: We added a missing reference: "Jones RJ, Yellowlees D. Regulation and control of intracellular algae (= zooxanthellae) in hard corals. Philosophical *Transactions of the Royal Society B*, 352(1352): 457 – 468, 1997".
- Figure S1: We adjusted the height of the coloured box (drawn only as guide to the eye) in panel 'd' because in the original manuscript this box extended, erroneously in relation to the actual data points, from 10% to 95%. The correct extension goes from 25% to 95%, as it was correctly reported in Table 2.

85: "…means that we always assume that the reef habitat has a maximal capacity of hosting an equal proportion of massive and branching corals." Could you please unpack this statement a bit more? It's not clear to me how the conversion factors mentioned in the preceding sentence ensure equal proportions of the two coral growth morphologies.

Here we refer to the conversion factors proposed by Chancerelle (2000). These factors are meant to estimate the effective surface area occupied by a coral colony by taking coral shape into account. This effective surface area is estimated with the formula: effective surface area = conversion factor x area on which colony is seated. For example: 1 m^2 of planar area corresponds to 11.86 m^2 of effective surface occupied by branching corals. This is why, by multiplying the planar surface area of reef habitats by these conversion factors, one can account, in roughly equal proportions, for the two most common coral morphologies in the computation of the regional carrying capacities. Admittedly, such morphologies might not always occur in exactly equal proportions in the regions of interest, this is why we specified that it is our assumption "that the reef habitat has a maximal capacity of hosting an equal proportion of massive and branching corals". Albeit with this caveat, this approach of estimating coral carrying capacities is more realistic than just considering the planar reef habitat area, as determined by Chancerelle (2000). To clarify this point we revised the relevant text (lines 112 – 116) as follows: "This approach for calculating coral carrying capacities allows us to account for the contribution, in roughly equal proportions, of the two most common coral morphologies. In reality, these morphologies may not always occur in equal proportions. Albeit with this caveat, a previous study (Chancerelle, 2000) determined that this approach produces more realistic estimates than simply considering planar habitat areas".

I also wonder whether this assumption of equal proportions is also one that could be relaxed (perhaps based on region-specific relative species abundance data) to test the robustness of findings.

Unfortunately, this assumption cannot be relaxed because our model does not resolve individual species. Our model is designed to describe the mean change of a trait with the aim of capturing the coral community as a whole acclimating entity.

110: missing 'cm' after 10 and 0.83

The unit of G_{max} is actually correct. In our model, the coral growth rate is quantified as "fold change per year", that is, "per year". Actual extension rates are measured as "cm per year", but we used these measurements as a general reference for constraining G_{max}, which is assumed to reflect the maximum growth rate of an assemblage of mixed coral types (massive and branching, as we specified in the original text) at optimal temperature, as expressed in Eq. 7. To avoid confusion on this aspect, we have specified that the maximum growth rate in our model is expressed in terms of fold change per unit of time (line 138).

152: "…host to control the nutrient flux. The latter ensures that the symbiont population never exceeds the hosting capacity of the corals." The authors later mention eutrophication as a potentially important additional factor to consider in the Discussion, but it could be useful to point out here how this assumption can be violated: data suggest that eutrophication can elicit a breakdown in the coral-zooxanthellae mutualism, whereby uncontrolled symbiont growth can actually depress coral growth (e.g., Gil, MA. "Unity through nonlinearity: A unimodal coral-nutrient interaction". Ecology 2013). It's outside the scope of this paper to speculate too much on potential indirect environmental effects that could interact with coral acclimation to rising temperatures, but it is also interesting to consider that eutrophication is often driven by runoff events that can increase in magnitude and frequency with warming, but in a region-specific manner - perhaps a cool future extension of this model.

Thank you for raising this interesting point. Following your suggestion, we have added the following text (lines 180 – 183): "In nature, eutrophication can concur with temperature to induce a breakdown of the symbiotic relationship through uncontrolled symbiont growth (Gil, 2013). These synergistic effects, however, are beyond the scope of our study".

201: 'a shifts' should be 'an increase'

Corrected (line 234).

221: "However, given that a potential reef habitat represents a snapshot of the total area that could be covered by corals, we assumed that the mean of all observations of coral cover reflects, qualitatively, a measure of coral abundance in relation to a carrying capacity (i.e. in relation to the total amount of coral cover that the considered region can sustain)." From this statement, it's not clear to me how the region-specific coral carrying capacity was determined. Is the carrying capacity the mean of all observations of coral cover? This would seem biased in favor of a lower carrying capacity than reality, given the high disturbance frequencies and magnitudes of these systems. Please provide further detail.

We have calculated these regional carrying capacities based on potential reef habitats and coral surface conversion factors, this was explained in the subsection "Coral dynamics" (see original manuscript, lines 86 – 92). To avoid potential confusion, we added a reference to subsection "Coral dynamics" (lines 262 – 263) where the specialised reader can find the relevant explanations. Also note that we have revised the part of "Coral dynamics" related to these conversion factors in response to your point above.

261: "…we conducted model simulations without bleaching." It would be helpful to describe the motivation for the modeling without bleaching.

Thank you for this comment. To motivate the numerical experiments without bleaching more clearly, we reformulated the relevant text (lines 292 – 298) as follows: "In order to assess the effects of the

long-term trend of global warming, we conducted model simulations under the hypothetical absence of short-term (monthly) temperature fluctuations, i.e. without bleaching. By visually comparing the model results obtained with bleaching and without bleaching, one can also evaluate, albeit qualitatively, the impact of bleaching events. The experiments without bleaching were performed by forcing the model with the annual averages of environmental temperatures for the period 1955-2100. The results of these experiments are presented in S4 Appendix."

266: "After this phase, we introduced the temperature forcing described in the previous section (for the period 1955–2100) in order to produce…" Should 1955 be 2000, since this would be after the previously mentioned phase of 1955-2000?

The cited text refers to the model spin-up phase, which is required to remove numerical artefacts typically occurring at the beginning of the simulations due to non-linearities in the model equations. During this phase the model is run for 2000 years with fixed temperatures so that the model can reach an equilibrium. As fixed temperatures, we considered the average temperatures between 1955-2000 in the different regions. We clarified this by modifying the relevant paragraph as follows (lines 302 – 309): "We first consider a spin-up phase of 2000 years during which we let the model dynamics evolve under fixed temperature (equal to the average temperatures of the period 1955 – 2000 in the three regions, which are 25.90 °C, 28.45 °C, and 27.57 °C for, respectively, the Great Barrier Reef, the South East Asia, and the Caribbean). This spin-up phase removes numerical artefacts typically occurring at the beginning of a simulation due to non-linearities in the model equations. After this phase, the model has reached an equilibrium and we then introduce the temperature forcing for the period 1955 – 2100 to produce the actual long-term model results".

Reviewer 2

This paper presents a model to explore a crucial topic in coral reef population ecology: the capacity for acclimatization in response to climate change. I was particularly impressed by the careful approach to model parameterization while maintaining generality.

Thank you for the positive words about our study.

My most substantive concern is that, to truly quantify the role of acclimatization, the paper needs to compare the results Fig. 3 to the case without any acclimatization (N=0). Without this comparison to isolate the effect of acclimatization, the authors cannot make conclusions about the specific role of acclimatization in their outcomes (e.g., lines 307-308), which is the primary goal of this paper. This should be a relatively straightforward comparison to do with the existing model.

Thank you for this suggestion. We have included runs without acclimation under RCP 2.6 (shown as black lines in the new Figure 3). The results of this test confirm our general conclusion that current rates of acclimation are insufficient to help corals to cope with increasing temperature. We have thus added the following sentence (lines 390 – 391): "The model runs conducted without acclimation confirms that the capacity of corals to respond to increasing temperatures is generally low." Additionally, we included the motivations for this test in subsection "Simulations and sensitivity analysis" (lines 325 – 331).

For the model structure, it took me a while to understand why the benefit of the plastic trait U, the energy investment in symbiosis, appears in the coral growth function (in dC/dt) rather than the symbiont dynamics (dS/dt). If one interprets this trait as physiological responses that bolster the symbionts during marine heat waves (e.g., heat shock protein up-regulation; as described on lines 198-201), then one would expect the benefit of greater U to come in through lower symbiont mortality during marine heat waves. I think the reason why the authors chose the approach they did is because they needed both the costs and benefits of U to be in the same function in order to have one function for taking the derivative to calculate the selection differential that determines the trait change dynamics (dU/dt); the costs do belong in the coral dynamics due to energy investment effects, so then the authors put in the benefit in terms of coral benefits from symbionts. Which I can understand from a tractability standpoint, but irregardless of whether this or another explanation is the right one, the authors need to clarify why they're taking the approach they do when they first develop the model (in the Coral dynamics section of the Methods), and discuss the potential effects of this assumption (e.g., the effect of benefits occurring continuously rather than in pulses during marine heat waves, and what types of acclimatization that does and doesn't represent) in the Discussion (somewhere around lines 402-405 where they're discussing phenomenological vs. mechanistic modeling approaches might make sense).

In nature, corals are in control of the symbiotic relationship, this is why we decided to take the perspective of the corals in our study by considering the energy invested by corals as the plastic trait *U* (thus *U* appears in the coral equation). Corals invest energy into the symbiotic relationship in order to "harvest" photosynthetic products from the symbionts. The benefits of investing energy into the symbiotic relationship (represented by coral gross growth, positive term in Eq. 4) do manifest themselves in pluses following heat waves (see new Figure in S6 Appendix, which we also present below in one of our responses to Reviewer 3, showing the two main components of coral fitness over time: gross growth and cost of symbiosis). The benefits of the symbiotic relationship for corals change when the physiological responses that bolster the symbionts are activated by the corals during heat waves. However, the new Figure in S6 Appendix shows that benefits and costs are nearly anti-reciprocal (because of the acclimation dynamics nature of our modelling approach, according to which the trait "moves" to values that optimise fitness) and thus cancel each other out, producing a continuous, smooth dynamics in the coral trait (Figure 3).

If at all possible, it'd also be great to see a functional sensitivity analysis comparison to a model structure where the benefits accrue in the symbionts rather than the coral, but I understand that this might not be mathematically tractable.

A sensitivity analysis comparing our results to results obtained with a model in which the benefits of symbiosis are accrued through the symbionts would be impractical because it would require profound changes in the model functional dependencies. These modifications would produce a very different model structure, which would then require a new modelling study. Another important aspect is that, in nature, the benefits of the symbiotic relationship between corals and algae are clearly not accrued through the symbionts. As we explained in the manuscript, corals are in control of this association and it is well established that corals receive most of the benefits of this association (more than 90% of the carbon fixed by the algae is translocated to the coral host) whereas the symbionts grow in a highly nutrient-limited environment under the coral epidermis (see for example, Falkowski et al. 1984 or Muscatine et al. 1984). Because of these reasons, this association has been even defined as “controlled parasitism” or “producer within consumer” (see: Wooldridge, 2010: Is the coral-algae symbiosis really ‘mutually beneficial’ for the partners? BioEssay, 32(7): 615 – 625).

For the model analyses, in addition to adding an analysis without acclimatization for comparison, the paper would be stronger if the authors had the sensitivity analysis (currently in the appendix, Figs. S6-S9) in the main text, as it recognizes uncertainty in the parameterization and leads to some of the more interesting conclusions about what most influences acclimatization potential. This doesn't have to be all four sensitivity analysis figures; the ones with bleaching are more relevant than the ones without, and the results were pretty consistent across climate scenarios so the authors could chose one climate scenario to illustrate the sensitivity analysis in the main text and put the remaining RCPs in the appendix. This would be even better, and the results presented more simply, if the authors took a more formal approach to sensitivity analysis, whether as a local sensitivity analysis or global sensitivity analysis as described in: Cariboni, J., Gatelli, D., Liska, R., & Saltelli, A. (2007). The role of sensitivity analysis in ecological modelling. Ecological modelling, 203(1-2), 167-182. plus see: Harper, Elizabeth B., John C. Stella, and Alexander K. Fremier. "Global sensitivity analysis for complex ecological models: a case study of riparian cottonwood population dynamics." Ecological Applications 21.4 (2011): 1225-1240 for some different ways to visualize global sensitivity analysis outputs.

Thank you for these suggestions. We have moved some of the figures showing the sensitivity analysis of the model with bleaching in the main text. Specifically, former Figures S6 and S7 appear now in the main text as Figures 5 and 6. Please note that we have revised the Discussion section substantially also in response to other reviewers’ comments.

Three points on the motivation for this modeling exercise in the Introduction:

1. While the Abstract and Author summary both start with establishing the threat that climate change poses to tropical coral reefs before getting into responses to climate change via acclimatization and adaptation, the Introduction launches straight into those responses. Before this, at the onset of the Introduction, the authors should have a short paragraph that sets up the impact climate change has on corals (with relevant references) before getting to acclimatization and adaptation potential.

Thank you for pointing this out. We have added a whole new paragraph at the beginning of the Introduction (lines 2 – 15).

2. For the motivation of focusing on acclimatization vs. genetic adaptation, I disagree with the authors' claim that that the long generation times of corals make evolution less relevant (lines 12-14, and repeated elsewhere: 3rd sentence of the Abstract, lines 67-69, 193-195, and 370-371), as evidence has now been mounting for decades that evolution can occur on ecological time scales (this body of research even now has its own field, eco-evo dynamics; see, e.g. Schoener 2011, Science 331:426-429). Even though corals are long-lived as the authors say, a selective sweep takes only one extreme event, such as a bleaching event, for gene frequencies to shift, and the symbionts within corals, which can also evolve, have very high turnover rates. Really, acclimatization vs. genetic genetic

adaptation is a false dichotomy: rather than either/or, both have the potential to play a role in future coral dynamics under climate change, and there's no need to claim one as unimportant to say the other is important. Instead, the authors could just make the point that both acclimatization and genetic adaptation might play a role in future coral persistence, and while a few models have quantified evolutionary capacity, quantifying acclimatization capacity as well can help further understand overall coral adaptive potential. Note that, once the authors have established the motivation for why one might look to quantifying acclimatization capacity in the Introduction, there's no need to repeat the motivation elsewhere in the manuscript (e.g., lines 66-69, 193-195).

Thank you for bringing up these points, we fully agree. We have reduced the emphasis on the contrast between acclimation and genetic adaptation in both Abstract and Introduction (lines 29 – 31) by reformulating the relevant text as follows: “However, given the long generation time of most corals, acclimation via phenotypic plasticity could be an important mechanism by which these organisms can cope with global warming”. We also modified a related sentence in the Models and methods section (lines 89 – 90) as follows: “Since our study is focused on coral acclimation as a mechanism for counteracting global warming…”. We removed the mentioned repetitions.

3. In both the Introduction and Discussion, I think there's a missed opportunity here to connect to the idea of "pre-exposure" (also called pre-conditioning, stress-hardening, and induced acclimatization, among other names) that is gaining interest in the field of coral reef restoration as a way to promote the likelihood of outplanted corals persisting under future climate change. See Chapter 3 on Physiological Interventions of the 2018 National Academies report on "A Research Review of Interventions to Increase the Persistence and Resilience of Coral Reefs", available at:https://www.nationalacademies. for a review of this approach. Quantification of acclimatization potential, as is done in this paper, can help inform the capacity for this management intervention to affect coral persistence under future climate change. In addition, might the model developed here provide a framework for the types of decision-support tools described in the second report linkedAcci above (on "A Decision Framework for Interventions to Increase the Persistence and Resilience of Coral Reefs"), which can help managers navigate whether and how to implement interventions like pre-exposure?

Thank you for bringing this to our attention. We have added the following sentence in the Discussion (lines 500 – 503): “Our current results, however, provide preliminary indications for the design of new management strategies aiming at increasing the resilience of coral reefs based on the specific acclimation capacities of coral communities we estimated in the different regions.”

The model could use greater clarity in presentation in a few places. In particular, somewhere in the Bleaching subsection of the Methods, it'd be helpful to have a mathematical expression for the implementation of bleaching with relevant parameters defined. Are the authors essentially having discrete bleaching events within the continuous-time model, such that this is a pulse-impulsive or semi-discrete model as described in: Mailleret, L., & Lemesle, V. (2009). A note on semi-discrete modelling in the life sciences. Philosophical Transactions of the Royal Society A: Mathematical, Physical and Engineering Sciences, 367(1908), 4779-4799?

The simulation of bleaching is implemented simply as a reduction in symbiont abundance when environmental temperature exceeds the optimal temperature for coral growth. To account for region-specific variabilities in bleaching responses, we used observational data to empirically determine the percent of symbiont abundance reduction in function of regional excess temperatures. These data are presented in full in Table 2 and S1 Appendix.

*In addition, equations (1)-(6) would be clearer if all state variable dependencies were consistently specified for all functions, e.g. F(U,S,C) instead of F(I, kappa*E,C) in eq. 1, K_S(C) instead of K_S in eq. 2, and kappa(S)E(U) instead of kappa*E in eq. 4 (with these state-variable-dependency specifications elsewhere in the text wherever relevant), perhaps with temperature T dependency specified where relevant as well.*

Thank you for pointing out this aspect. In the revised manuscript, we specified all the functional dependencies in the set of equations (1 – 6) and, to further improve readability, we included multiplication symbols (as dots).

Additional comments:

First sentence of the Introduction on line 1: in addition to adaptation and acclimatization, there is a third commonly-invoked response that organisms might have to climate change, range shifts, which tend to get discussed less in the context of coral reefs but are one of the most frequently-observed responses to climate change across systems: Parmesan, C. (2006). Ecological and evolutionary responses to recent climate change. Annu. Rev. Ecol. Evol. Syst., 37, 637-669.

Thank you for pointing this out. We agree that range shift is an important mechanism of coping with a warming ocean. Interestingly, for sessile species like corals, range shifts are constrained by dispersal during their larval stage (Pinsky et al. 2020: Climate driven shifts in marine species ranges: scaling from organisms to communities. *Ann. Rev. Mar. Sci*.) which movements may be strongly influenced by the prevailing currents. A relatively recent meta-analysis across many marine species found that ocean flows can both facilitate and hinder range shifts depending on their directional agreement with spatial gradients of temperature (Molinos et al. 2017: Ocean currents modify the coupling between climate change and biogeographical shifts. *Sci. Rep.*). When larval dispersal is the dominant mechanism, then colonisation also requires the growth and maturation of settled juveniles in the new habitat. The topic is fascinating but clearly beyond the scope of our study. Nonetheless, as you suggested, we have revised the relevant text (lines 16 – 19) as follows: "Corals can respond to global warming in different ways, including by moving to more favourable habitats, by genetic adaptation, or by acclimation. For sessile species like corals, range shifts are constrained by dispersal during their larval stage, the movements of which are strongly influenced by the prevailing currents (Pinsky et al. 2020)."

Line 60: "adaptive dynamics" invokes a specific invasion analysis approach to modeling evolutionary change, please use a different phrase here.

Agreed, we replaced "adaptive dynamics" with "acclimation dynamics" throughout the manuscript.

Line 65: "plastic trait" would be a more precise label than "adaptive trait".

Agreed, we replaced "adaptive trait" with "plastic trait".

Lines 161-171 are repetitive to the Introduction, and spending this amount of space on DHMs in the Methods might mislead a reader into thinking that the authors are going to take a DHM approach. I would start with the approach taken here, then briefly justify it.

Agreed, we removed the description of DHM and DHW from this paragraph.

Line 209: this could be more clearly framed as an assumption that plasticity is adaptive in the case of this model, as in reality plasticity is not always adaptive, especially when human-driven environmental change leads to "evolutionary traps".

We fully agree on the fact that plasticity is not always adaptive and may, in some cases, lead to evolutionary traps, but the "adaptive plasticity hypothesis" that we cited in this context is clearly related to positive adaptation.

Section on "Simulations and sensitivity analysis": what are the initial conditions for the simulations?

Thank you for spotting this out. The initial conditions were chosen as follows:

$C(t=0) = 0.75 \times K_C$ cm^2

$U(t=0) = 5 \times 10^{-7}$ energy cm^{-2} month^{-1}

$S(t=0) = 10^{-3}$ cells

As explained in the revised manuscript (lines 300 – 309), our simulations start with a spin-up phase of 2000 years during which the model is forced with fixed temperatures in order to reach equilibrium. The fixed temperatures are chosen as the average temperatures of the period 1955 – 2000 in the three regions, which are 25.90 °C, 28.45 °C, and 27.57 °C for, respectively, the Great Barrier Reef, the South East Asia, and the Caribbean. After this phase, the model is forced with variable environmental temperatures (from 1955 to 2100). Therefore, the initial conditions for the three state variables refer to the beginning of the spin-up phase. Since we do not have any information about the abundances of corals and symbionts in the regions of interest 2000 years prior 1955, we adopted a conservative approach by setting the initial coral abundance to 75% of the coral regional carrying capacities (which we estimated from suitable coral reef habitats). This is the only initial condition chosen according to data and qualitative biological arguments. The initial conditions for *U* and *S* were chose based on technical constraints in order to ensure that the model results are at equilibrium throughout the simulations and to avoid numerical crashes. Note, however, that the functional relationships between corals and symbionts in our model (e.g. the amount of symbiont abundance per coral abundance, K_{smax}, used to calculate the symbiont carrying capacity, see section "Symbiont dynamics") are based on observational data. Therefore, despite the caveats involved, we are confident that, during the spin-up phase, the model results evolve towards realistic abundance levels for both symbionts and corals. See also Figure 2 in which a series of model results are compared to coral cover data. We added this information in the revised manuscript (lines 301 – 323).

Lines 275-285: this text is repetitive to the Introduction.

We prefer to keep this text (which is formulated in a slightly different way than in the Introduction) because we find it relevant to emphasise here the important gap in knowledge about the speed of coral acclimation just before we report our first estimates of these speeds in the regions we analysed. Another reason is also that some readers may be only interested to read the results and so a brief introduction into the topic may be useful in this section.

Line 333: typo in "important".

Corrected

Line 349-350: this sentence is a fragment.

Thank you for pointing this out. We completely removed this sentence because it was out of context in that paragraph.

Lines 400-401: "theory of adaptation" should be "theory of acclimatization"; a generalized theory of adaptation would have both genetic and plastic responses.

Corrected.

In addition, this discussion of mechanistic vs. phenomenological approaches to modeling might benefit from the example of dynamic energy budget models as a mechanistic approach in contrast to the phenomenological approach here; note that DEB models have been applied to corals: Cunning, R., Muller, E. B., Gates, R. D., & Nisbet, R. M. (2017). A dynamic bioenergetic model for coral-Symbiodinium symbioses and coral bleaching as an alternate stable state. Journal of Theoretical Biology, 431, 49-62. Muller, E. B., Kooijman, S. A., Edmunds, P. J., Doyle, F. J., & Nisbet, R. M. (2009). Dynamic energy budgets in syntrophic symbiotic relationships between heterotrophic hosts and photoautotrophic symbionts. Journal of Theoretical Biology, 259(1), 44-57.

Good point, thank you. We added this in the Discussion section (lines 497 – 500).

For the discussion of the sensitivity analysis, note that a potential implication of the high sensitivity to coral growth (lines 406-414) could be that local management approaches that might buffer coral growth, such as control of the runoff of pollutants that affect coral growth as reviewed in:

Fabricius, K. E. (2005). Effects of terrestrial runoff on the ecology of corals and coral reefs: review and synthesis. Marine pollution bulletin, 50(2), 125-146.might then increase acclimatization potential to global climate change.

Thank you for pointing this out. We have now linked the sensitivity of our model results to management aspects and added the suggested reference (lines 482 – 484) as follows: “This suggests that local management policies aiming at reducing nutrient runoff and pollution could be an effective strategy for “controlling” coral growth (Fabricius, 2005) and, thus, for mitigating the decline of coral communities (see Fig. S9).”

I appreciate that the authors discussed a few of the model assumptions throughout the Discussion. Another assumption to consider discussing is whether and how the model represents the potential for constraints to plasticity or acclimatization potential.

We are not sure if we fully understand this point. If the reviewer considers this point critical, we would appreciate some clarification.

Reviewer 3

The authors model corals acclimating to increasing temperatures, where corals acclimate by changing the amount of energy they invest in the symbiosis. Energy investment can potentially make up for symbionts lost due to bleaching, by increasing the per-symbiont benefit of the symbiosis to the coral. The authors build a differential equation model that tracks the growth and energy investment of a single coral and symbiont. They investigate corals from three regions (the Great Barrier Reef, South East Asia, and the Caribbean) under three different temperature scenarios, and find that corals on the Great Barrier Reef are best able to use acclimation to survive, but none of the three regions' corals could survive via acclimation alone in the hottest warming scenario.

Coral acclimation is a topic of great interest for conservation, and the authors' focus on using incorporating real warming and trait data to understand the ability of coral acclimation to preserve reefs is likely to be of interest to many readers. The model and results are presented very clearly, and the authors do an excellent job of explaining the model's purpose and future uses.

We thank the reviewer for the positive words about our study.

There is a potential issue in the function for the temperature-dependent growth rate of the symbiont. The authors model the maximum symbiont growth rate as increasing exponentially with temperature. This relationship comes from studies across multiple species, where the maximum growth rate over all species at a particular temperature follows this pattern. However, individual species's growth rates probably do not follow this pattern (the Baskett et al. paper they cite models them as decreasing sharply after a certain temperature). For the model, this seems to imply that the coral is swapping out its symbionts every time the temperature changes, to always have a species that has the ability to achieve the maximum growth rate a particular temperature. I know the model is intended to provide information about populations, but since the differential equations so closely resemble the interaction between a single coral and symbiont, I think there are probably some consequences of modeling symbiont growth this way. In particular, the symbiont growth rate may be lower than is modeled (because most symbionts will not actually be growing at this rate). I think this decision and its consequences should be discussed.

Thank you for this comment. We are aware of the fact that symbiont growth varies with species and that the sensitivity to temperature is also species-specific. However, here we have chosen to considered the symbiont community as a single (acclimating) entity. Additionally, the acclimation capacity of the coral community is considered here under a “best-case” scenario. The investigation of the effects of different combinations of symbiont species and their shuffling under changing environmental temperature are actually the object of our next modelling project, which is inspired by the results we presented here. To clarify this aspect, we have added the following text in the Discussion (lines 504 – 509): “Additionally, our modelling approach considers coral and algal communities as single entities, despite evidence shows that the thermal acclimation of symbionts could be species-specific and that harbouring mixed algal populations could constitute an ecological advantage for corals (Berkelmans & van Oppen, 2006). The effects of different combinations of symbiont species and their shuffling under changing environmental temperature are thus additional avenues for promising future research.”

Other comments:

I think it would be really helpful for gaining an intuitive understanding of the model to be able to see how the benefit of energy investment changes with temperature. A plot of the optimal investment for one of the warming scenarios would be nice, if that is possible to make.

In our model, the benefits of energy investment are represented by coral gross growth (the positive term in Eq. 4). In the Figure below, we plotted benefits and costs (i.e., gross growth and costs of symbiosis) over time for the three regions and the respective warming scenarios. The costs of symbiosis (orange lines in the Figure) represent the amount of gross growth that corals invest in the symbiotic relationship. Investment into symbiosis, therefore, follows closely the trajectory of the

benefits received by the corals from the symbiotic relationship. When such benefits are minimal also investments are minimal and vice versa.

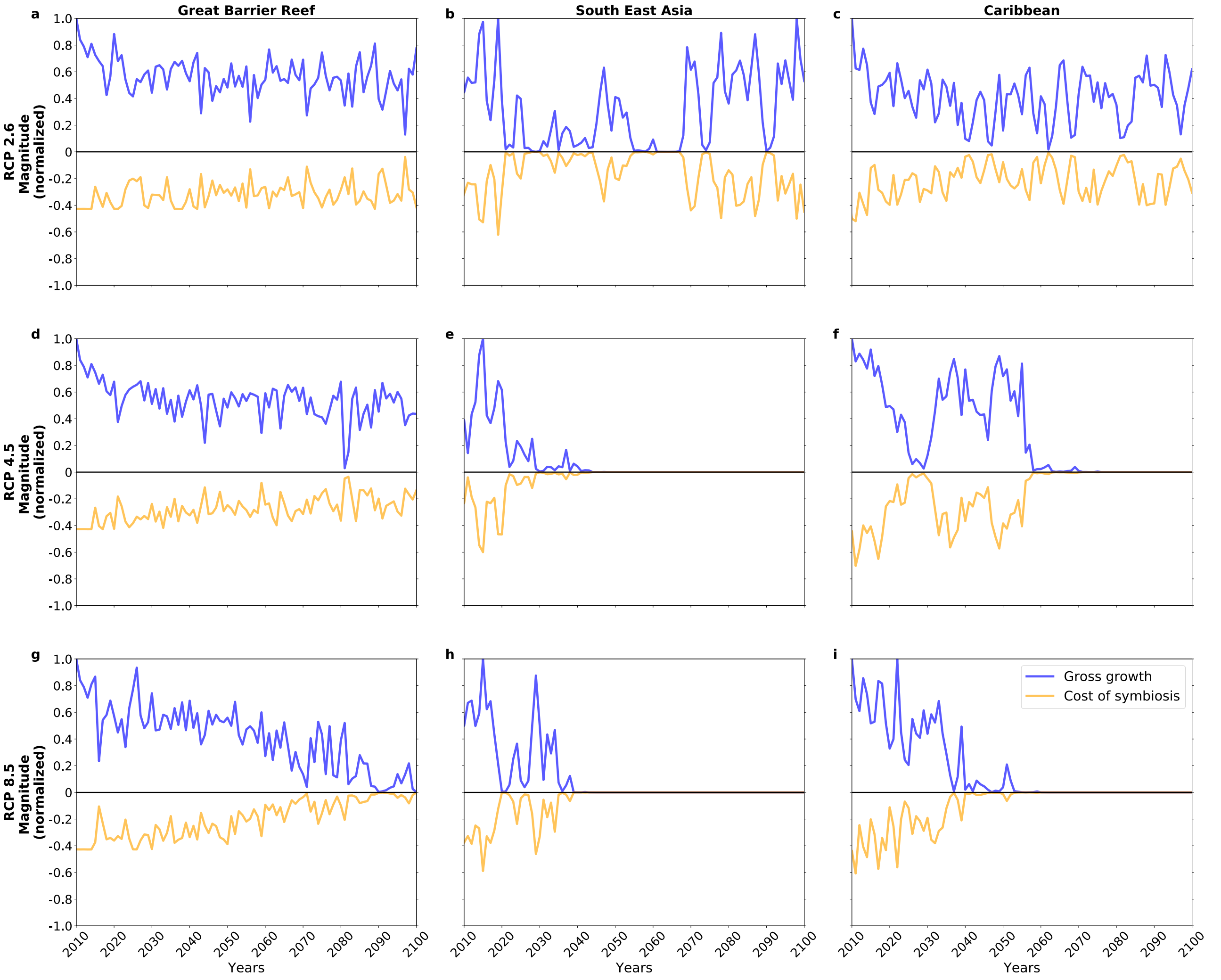

Optimal energy investment over time is represented by the percent change in the coral trait *U* (shown in the manuscript in Fig 3d-f). In our model, the trait *U* changes over time in the direction of increasing fitness. Therefore, at any given time, the trait *U* is such that fitness (that is, net coral growth, Eq. 4) is highest or 'optimal' under the given environmental conditions. To clarify these aspects, we added the Figure above in S6 Appendix. Additionally, we added explanatory text in the Results section (lines 380 – 389). See also the new text in S6 Appendix.

The authors refer to the model as an adaptive dynamics model, which I think might be confusing to readers. To me adaptive dynamics is a technique for modeling evolution, and this model is important because it does not include evolution. Also, adaptive dynamics seems to me to require selection and some kind of inheritance mechanism. Possibly there is an argument that some sort of selection is present (maybe between polyps?). I think it would also be reasonable to say that this is a sensible acclimation rate to assume for other reasons. (It seems like a pretty sensible way to try to match your environment!)

Thank you for pointing this out. We replaced **"**adaptive dynamics" with "acclimation dynamics" throughout the manuscript.

It seems to me that by changing the strength of the symbiotic feedback (beta) and and the coral exponential cost parameter (r), it is possible to scale energy (and thus rate of acclimation) values. I don't think this is a problem for the model, but I do think this means it would be helpful to give some intuition for what an energy investment of a particular amount means to the model coral (maybe something like how much of its growth is it investing in the symbiont?), and why the beta and r values were chosen.

Please see our response to your point above and the Figure (S10 in revised the manuscript) we show above in support of our explanations.

The temperature-dependent host growth rate is derived from coral distribution data. Intuitively, it makes sense that if no corals can grow at a temperature, their growth rate at that temperature is zero. I think a sentence or two explaining how to get from the coral distribution to the growth rate function in the nonzero growth rate case would be nice.

The topic of coral growth is discussed in detail in the section “Coral dynamics”, which we have now revised also in response to other reviewers’ comments. In light of your comment, we added the following sentence (lines 132 – 134) for clarification: “If environmental temperature falls away from these tolerance ranges then coral growth will be zero and the abundance of the community will decline.”

I just skimmed the code, but it looks like some small terms are being added to the denominators of some functions to prevent division by 0. If so, it would be good to mention that in the methods section.

Thank you for taking the time to look at the code, we really appreciate that. We have added the following sentence in the section Simulations and sensitivity analysis (lines 300 – 301): “The mathematical model was coded in Python. Small terms were added to the variables in the denominators in order to prevent divisions by zero.”
