# Supplementary material for: Modelling the acclimation capacity of coral reefs to a warming ocean
Source: PLoS Comput Biol. 2022 May 9;18(5):e1010099. doi: 10.1371/journal.pcbi.1010099 (PMC9119535; doi:10.1371/journal.pcbi.1010099)
Supplement: Attachment — Submitted filename: Responses20220409.pdf [file pcbi.1010099.s008.pdf]

**Point-by-point replies to reviewers**

We would like to thank all reviewers for the additional suggestions and explanations. We greatly appreciate their constructive spirit and the time they dedicated to our study. These additional comments have helped us to further improve the clarity of our manuscript. Below we respond, point-by-point, to the comments (quoted in italics) and we specify the revisions implemented in the manuscript. The lines we cite in the responses refer to the revised manuscript with tracked changes.

**Reviewer #1:**

*I appreciate the efforts the authors put into their revisions in response to my and the other reviewers' comments. I believe the manuscript is clearly improved. The issue I have with this version is that I think the authors can and should be more up front about the fact that while they motivate this work around the need to understand 'coral acclimation', their model - as they acknowledge in the first paragraph of the discussion - deals with changes in phenotypes at the community level, something one could define as 'acclimation at the community level', I suppose. I struggle with this, because - again, as the authors acknowledge - species turnover is unavoidably a potential major mechanism underlying such 'acclimation at the community level', though this is not a mechanism one would think of as falling into the category of 'acclimation', since we (and the authors, in the Intro), tend to define acclimation at the organismal/species (not community) level. I think it would be better to be much more up front about this modeling study's limitations with respect to assessing the capacity for 'coral acclimation' to respond to climate change. I'd recommend clarifying in the Abstract and Intro that this study focuses on the community level (and, thus, cannot isolate the effects of organismal adaptation), at which species turnover can (and, according to recent studies by Terry Hughes and colleagues working in the GBR, likely does) play a substantial role in shaping community phenotypic shifts in coral responding to environmental changes over time. For example, in the Abstract and Intro, the authors discuss the capacity for corals to acclimate via phenotypic plasticity, but to not confuse readers about the scale of inquiry for the actual study, I'd argue it would be more appreciate to discuss 'coral community acclimation', or some other term, and clarify that what we traditionally think of as acclimation or adaptation cannot be explicitly examined with the proposed model and that species turnover could be a critical driver.*

Agreed. We revised the Abstract and the Author summary. We also specified in the Introduction that the speed of coral acclimation is estimated at the community level (line 48) and that the model does not resolve single species or individual organisms (lines 56 – 57, 60 – 62). To clarify these aspects, see also the changes we implemented in Models and Methods (lines 232 – 237) and Discussion (lines 424).

**Reviewer #2:**

*The reviewers have mostly addressed my previous comments, especially with the added simulations without plasticity as a baseline for comparison, as well as the added figure S6 to illustrate the benefit and cost dynamics. However, some lingering issues remain from the new changes and unresolved previous comments (all line numbers refer to the revised manuscript without tracked changes):*

*1. While I agree with Reviewer #1's point about shifts in community composition, I disagree with the changes made in response: with the model setup, a shift in community composition would change parameters related to coral type, such as Topt and Gmax (as mentioned on line 452-453), perhaps with some changes in N too if different coral species had different plasticity levels, but I expect that this is swamped by the other differences. Therefore, instead of adding the claim that the model captures changes in community composition (lines 345 and 403-404), I support the original reviewer feedback of adding this as a caveat, i.e. recognizing that the acclimatization rates might be over-estimated because the model doesn't capture the effects community shifts on coral cover dynamics.*

Agreed. We corrected these aspects by implementing revisions in the Abstract, in the Author summary, in the Introduction (48, 56 – 57, and 60 – 62), in the Models and Methods (lines 232 – 237), and in the and Discussion (lines 424).

*I also support Reviewer #1's suggesting to include sensitivity to a couple of different values for the percent of each coral type present among the sensitivity analyses, which the authors had declined to do.*

The proportion of the two most common coral morphologies was used to estimate the carrying capacities $K_C$ in the different regions. Therefore, we explored the model sensitivity with respect to changes in $K_C$. These sensitivity analyses were already presented in the original submission and are now shown in Figures S3 and S4 in S5 Appendix: Sensitivity analysis. These analyses show that $K_C$ is among those parameters which changes (within $\pm 25\%$) have little impact on the model results.

*2. With moving some of the sensitivity analysis results to the main text in Figs. 5-6 as I had suggested, the authors also need to add Results text about these figures in the main text, which is currently missing.*

Thank you for spotting this out. We added text describing the results of these sensitivity analyses (lines 407 – 414).

*3. While the authors indicate their agreement with my previous point about acclimation and genetic adaptation both playing a role in coral dynamics, and the false dichotomy inherent to presenting these as an either/or, the changes to the text do not reflect this: the framing language of "however", "alternately", and "although", as well as the continued mention of generation times (which, as I mentioned before, do not necessarily mean evolution is slow given the capacity for selective sweeps) and ecological vs. evolutionary time scales (which applies to macroevolutionary processes of speciation, not microevolutionary process of changes in gene frequency), on lines 29-31, 424-426, and in the abstract third sentence still imply that these are alternate, not co-occurring dynamics, and discounts the potential role of evolution. Please rephrase to recognize that both have the potential to occur, which can still motivate this study, as some studies have explored the role of genetic adaptation, but a mechanistic investigation into the role of acclimation is less well understood (with an eventual goal, hopefully, of including both to understanding their interaction, but understanding each in turn is a reasonable starting point and important to establishing their potential relative contributions before such integration).*

Corrected. We rephrased the relevant text in the Abstract, and removed the mentioned text from the Introduction (lines 27 – 31) and from the Discussion (lines 446 – 448).

*Also, in lines 36-38, note that the rolling-window approach to approximating adaptive and acclimation potential in these papers does not have an assumed-in bleaching threshold as implied here, but rather derives the threshold from the climatology, in particular the most recent mean of maximal monthly temperatures.*

Corrected (lines 37 – 38).

*4. The added text about stress-hardening on lines 465-468, a point that I had raised in my previous review, is too vague to be useful. Please either (a) be specific about stress hardening (using that term and defining what it involves) as a potential management application, including citations on this approach, and how this model might inform that approach, or (b) delete this addition. For option (a), note that the addition is out of place: it is a sentence about management implications in the midst of a paragraph about model assumptions. Therefore, if included, it should be moved to a more appropriate place in the discussion.*

Agreed. We followed your suggestion of removing the text (lines 488 – 491).

*5. For the data-based bleaching simulations: even if the parameters were estimated empirically, those parameters were somehow used in a mathematical expression (represented by code) to translate into their effect on coral dynamics. Please provide this information for full methods clarity.*

Agreed. We improved and completed the description of bleaching in the Methods section (lines 198 – 218 and Table 2).

*6. To clarify my earlier comment about adaptive plasticity: what this text (lines 225-236) needs clarity on is the idea that plasticity is always perfectly adaptive, as represented in the model, is an assumption and does not always occur in reality, as, in reality, plasticity can sometimes decrease fitness, such as in the case of evolutionary traps. The current text could be misinterpreted to mean that the idea that plasticity is always adaptive is a biological truth.*

Agreed. We specified that although in our model acclimation moves the trait in the direction of increasing fitness, plastic responses in nature can actually decrease fitness and thus be maladaptive (lines 244 – 245).

*7. For the addition about nutrient runoff and pollution effects on coral growth stemming from my previous feedback (lines 449), "controlling" doesn't make sense here; something like "buffering", "protecting", or (my preference) "mitigating anthropogenic impacts on" would work better (and why is this in quotes?).*

Agreed. We changed “controlling” with “mitigating anthropogenic impacts on” (lines 471 – 472).

*8. On line 394, do you mean "acclimate" instead of "adapt"?*

Corrected (line 416).

**Reviewer #3:**

*The authors addressed my comments, and there is just a small thing that I think should be added: I think in the methods the authors should say that the symbiont growth function comes from a curve of multiple species' thermal optima. The authors talk about modeling symbiont communities in the discussion, but I think it would be good to have this information about the function in the methods where the function is first described. Because the model partly has the purpose of being the "parent" of future models, it's likely that future readers will spend a lot of time with the methods section and would benefit from having all the information together.*

Agreed. We added this information in the description of symbiont growth (lines 165 – 167).

*I really liked the supplementary plots of the costs and benefits of the symbiosis. Figure S10 and all of section S6 helped me gain a better understanding of the model.*

Thank you for the positive comment.

** line 49: "coral's" should possibly be "corals'"?*

Corrected (line 46).

** line 386: "because of the acclimation dynamics nature of our modeling approach." I think removing "acclimation dynamics" from this phrase would make it clearer.*

Corrected (line 389).
